# Optimal design and biomechanical analysis of sandwich composite metal locking screws for far cortical locking constructs

**DOI:** 10.3389/fbioe.2022.967430

**Published:** 2022-09-27

**Authors:** Yuping Deng, Dongliang Zhao, Yang Yang, Hanbin Ouyang, Chujiang Xu, Liang Xiong, Yanbin Li, Wenchang Tan, Gang Huang, Wenhua Huang

**Affiliations:** ^1^ Department of Orthopedics and Traumatology, Integrated Hospital of Traditional Chinese Medicine, Southern Medical University, Guangzhou, China; ^2^ Guangdong Provincial Key Laboratory of Medical Biomechanics, School of Basic Medical Sciences, Guangdong Engineering Research Center for Translation of Medical 3D Printing Application, National Key Discipline of Human Anatomy, Southern Medical University, Guangzhou, China; ^3^ Institute of Biomedical Engineering, Shenzhen Bay Laboratory, Shenzhen, Guangdong, China; ^4^ Guangdong Medical Innovation Platform for Translation of 3D Printing Application, The Third Affiliated Hospital of Southern Medical University, Guangzhou, China; ^5^ State Key Laboratory of Chemical Oncogenomics, Drug Discovery Center, School of Chemical Biology and Biotechnology, Peking University Shenzhen Graduate School, Shenzhen, Guangdong, China; ^6^ Orthopaedic Center, Affiliated Hospital of Guangdong Medical University, Guangdong Medical University, Zhanjiang, China

**Keywords:** locking screws, sandwich structure, dynamic stabilization, high-cycle fatigue, screw optimization

## Abstract

In the interests of more flexible and less stiff bridge constructs to stimulate bone healing, the technique of far cortical locking has been designed to improve locked plating constructs in terms of stress concentration, stress shielding, and inhibition of issues around fracture healing. However, far cortical locking screws currently lack objective designs and anti-fatigue designs. This study investigates an optimization algorithm to form a special locking screw composed of various metals, which can theoretically achieve the maintenance of the excellent mechanical properties of far cortical locking constructs in terms of fracture internal fixation, while maintaining the biomechanical safety and fatigue resistance of the structure. The numerical results of our study indicate that the maximum von Mises stress of the optimized construct is less than the allowable stress of the material under each working condition while still achieving sufficient parallel interfragmentary motion. Numerical analysis of high cycle fatigue indicates that the optimized construct increases the safety factor to five. A high cycle fatigue test and defect analysis indicates that the sandwich locking constructs have better fatigue resistance. We conclude that the sandwich locking construct theoretically maintains its biomechanical safety and fatigue resistance while also maintaining excellent mechanical properties for fracture internal fixation.

## 1 Introduction

Research over the past 50 years has consistently demonstrated that controllable axial dynamic internal fixation systems effectively promote callus formation and improve the speed and strength of fracture healing (Goodship and Kenwright 1985; Kenwright et al., 1991; Claes et al., 1998; Panagiotopoulos et al., 1999; Uhthoff et al., 2006; Bottlang et al., 2010a; Richter et al., 2015). In the development of the fracture internal fixation constructs from Arbeitsgemeinschaft fuer Osteosynthesefragen (AO) to Biological Osteosynthesis (BO), blood supply protection and interfragmentary motion is preferred over absolute stability. Secondary bone healing is induced by interfragmentary motion in the millimeter range and can be enhanced by passive or active dynamization (Perren 1979; Claes et al., 1998; Duda et al., 2002; Hente et al., 2004; Zhang et al., 2012). The stiffness of a fixation construct is a principal determinant of fracture-site motion and thereby affects the mechanism and progression by which a fracture heals. The relatively high stiffness of fixation constructs may therefore suppress flexible motion to a level insufficient for optimal promotion of secondary bone healing (Bottlang et al., 2010b; Rodriguez et al., 2016; Plumarom et al., 2019). The advent of locked plating systems has provided a new strategy for dynamic stabilization, because the locked plating construct with fixed-angle locking screws does not require compression of the fixation plate onto the bone surface (Perren et al., 1973). The Far Cortical Locking (FCL) internal fixation system developed based on the locked plating system can achieve flexible axial fixation while retaining the stability of the internal fixation construct to a certain extent (Bottlang et al., 2009). The FCL construct can achieve controlled axial movement by bending screws that lock anchored in the plate and far cortical bone, but preserve range of motion at the proximal cortical bone. A study by Bottlang et al. claimed that in ovine models, a far cortical locking group had a 36% greater callus volume (*p* = 0.03) and a 44% higher bone mineral content (*p* = 0.013) than did the locked plating group (Bottlang et al., 2010a). A clinical study reported that distal femoral fractures were stabilized by the FCL construct, with a mean healing time of 16 weeks and an incidence of nonunion of 3% (Bottlang et al., 2014).

Currently, the FCL construct has been industrialized by several companies that commercialize orthopedic implant devices and is increasingly used clinically (Bottlang et al., 2014; Adams et al., 2015; Moazen et al., 2016; Kidiyoor et al., 2019). At the same time, in clinical practice, many surgeons can use standard locking screws to achieve the adoption of FCL technology by excessive drilling or slotting of the proximal cortical layer of bone. Many experts even claim that today’s FCL construct can be used as the “gold standard” for the treatment of distal femoral fractures (Bottlang et al., 2014; Plumarom et al., 2019; Wang et al., 2019). However, the flexibility of the fixation means the sacrifice of structural stability. Many scholars have put forward higher design requirements for the FCL construct: A study by Nahir et al. found that among all fracture fixation methods, the FCL construct had greater shear force than did the bicortical locking and non-locking structures, while the shear displacement is not conducive to callus growth (Habet et al., 2019). The FCL construct has a longer elastic element to reduce stiffness and provide a flexible fixation, but screw safety due to stress concentration and fatigue performance are worse than the external fixator attributed to the smaller screw diameter. During surgery, the adjacent cortical bone needs expanding to meet the requirements of FCL construct design, and the staggered and converging screw arrangement is implemented by 9° in FCL constructs to improve construct strength in torsion—this creates considerable challenges during surgery when working with a bone that has an irregular cross-section. The FCL screws are less safe and have a more significant fatigue fracture risk than do the locked plating screws that were reported in recent research (Deng et al., 2021). On the basis of these theoretical and clinically emerging concerns, several strategies to optimize the biomechanical properties of FCL constructs have been investigated. These strategies include optimizing FCL screw distribution, decreasing the plate elevation, and decreasing the plate span (Richter et al., 2015; Habet et al., 2019; Sarwar et al., 2021). While these strategies are effective for reduing structural safety hazards of FCL constructs to varying degrees, they also reduce their flexibility. What’s more, currently there is no research focusing on the optimization of high cycle fatigue performance of FCL construct. Although FE-based fatigue analysis is commonly used for reliability studies in engineering, it has been relatively rare to be applied in the biomedical fields.

Composite sandwich structure, as a new type of efficient and multifunctional structure, has been widely used in various fields such as architecture, aerospace and so on because of its high designability and outstanding mechanical properties. Through reasonable selection of materials and sandwich structure design, the mechanical properties of the material structure can be effectively improved while the volume or mass of the structure can be reduced (Sarvestani et al., 2018; Hayat et al., 2019). The concept of dynamic fixation to promote fracture healing is advocated in the current internal fixation treatment of fractures, which raises concerns about the safety and durability of implanted structures. Therefore, the use of composite sandwich structure to improve the statics and fatigue resistance of implant has a wide range of application prospects in the field of medicine.

In this study, calculation of a finite element numerical simulation was proposed for performing intelligent screening and optimizing of multi-working conditions for the locking screws of the FCL constructs. While optimizing the screw section of the FCL constructs, an optimization algorithm was proposed for forming a special composite metal sandwich structure locking screw made of titanium alloy and various metals. We used Isight platform independent programming for co-simulation and intelligent optimization, for static safety assessment with the allowable stress method, and for fatigue safety assessment through high-cycle fatigue analysis. What’s more, the reliability of the optimization scheme was preliminarily verified by biomechanical experiment and defect analysis. The newly designed composite metal sandwich locking screw can achieve elastic fixation, progressive stiffness, uniform load distribution, and parallel interfragmentary motion of the fracture end in the far cortical locking construct, while maintaining better fatigue resistance and torsion resistance to the internal fixation construct. This study provides a more objective digital operation basis and a more ideal structure design for the application of FCL construct. This may provide a new and reliable dynamic fixation method for clinical fracture treatment.

## 2 Materials and methods

**FIGURE 1:**
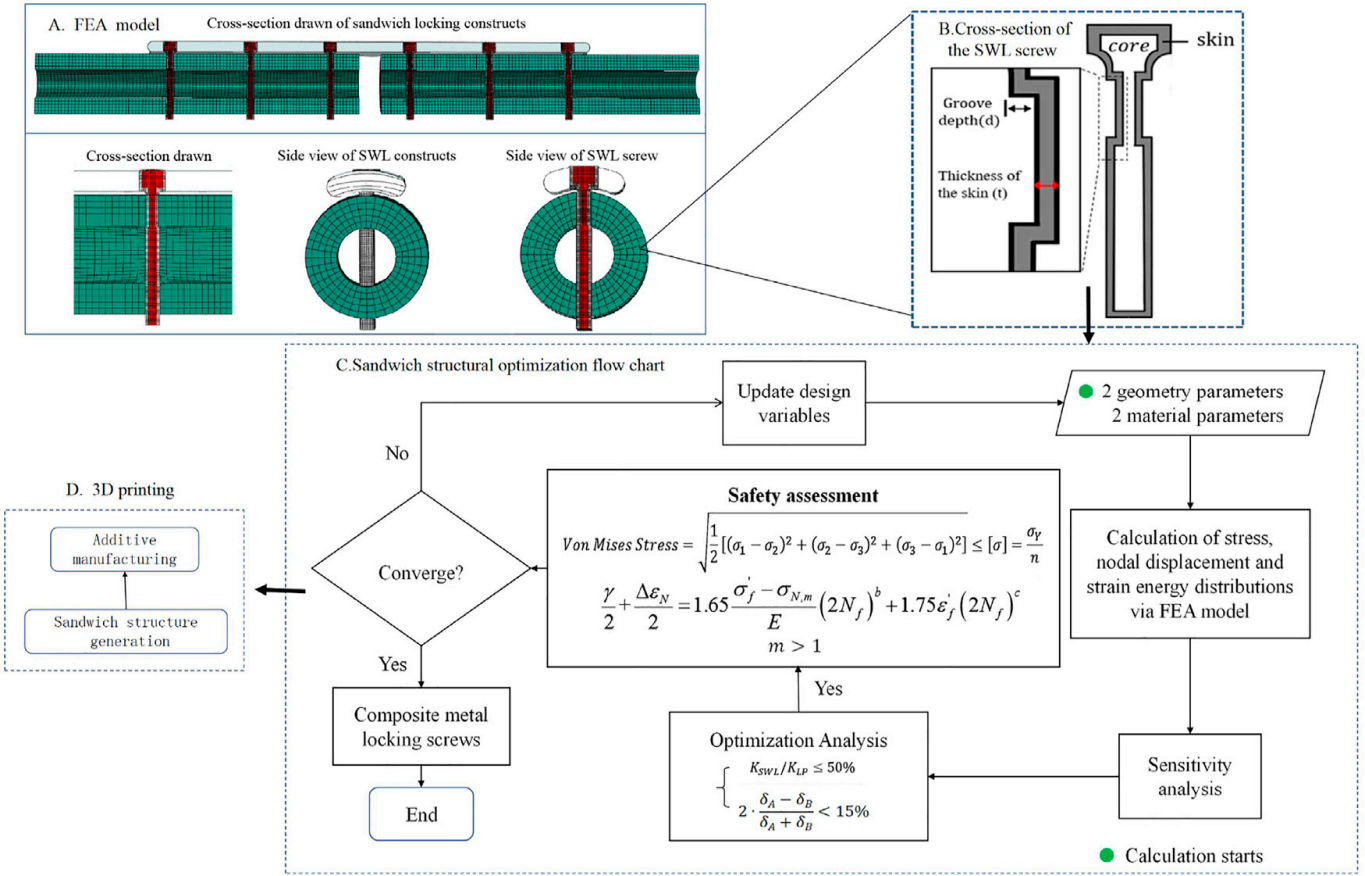
Steps for optimizing a sandwich composite metal locking screw. **(A)** Section view of optimized finite element model of sandwich locking (SWL) screw, and structure schematic diagram. **(B)** Schematic diagram of a cross-section of a composite metal screw with a sandwich structure. The core is a sandwich structure, the skin is the titanium layer structure on the surface of the screw, d is the depth of the groove of the screw at the proximal cortex, and t is the thickness of the protective structure of the surface titanium layer. **(C)** Flow chart of sandwich locking screw optimization for each design parameter, **(D)** Sandwich structure generation and additive manufacturing of the screws.

Our goal here is to develop a sandwich composite metal locking screw with tailored elastic modulus and morphology resulting in an optimal material selection and distribution that can achieve a reliable fatigue resistance while retaining the advantages of current FCL constructs: flexible fixation, uniform load distribution, progressive two-phase stiffening and parallel interfragmentary motion. Figure 1 illustrates the optimization steps for developing the sandwich composite metal locking screw.

### 2.1 Numerical model

**FIGURE 2:**
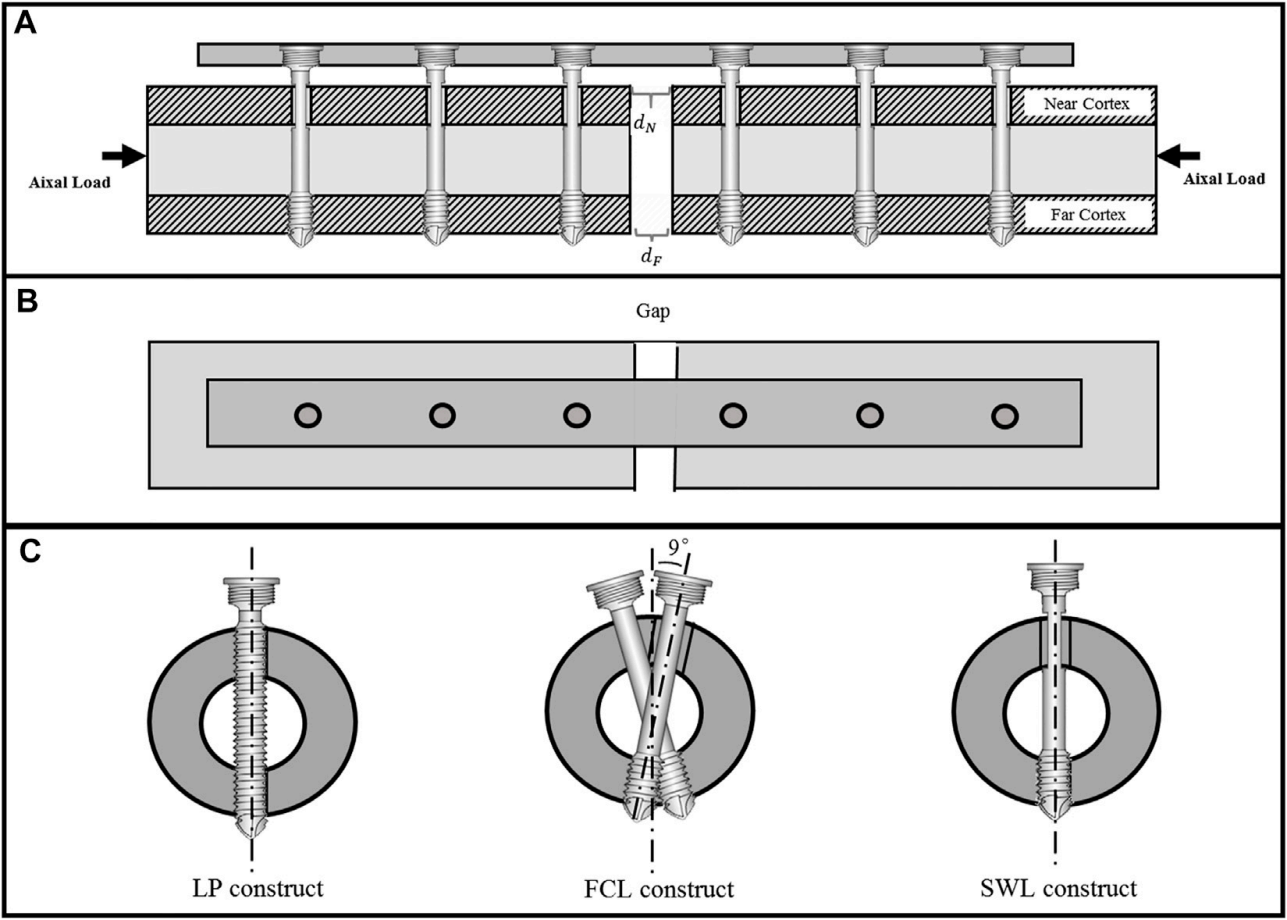
Schematic diagram of sandwich composite metal locking screw for far cortical locking construct. **(A)** Schematic diagram of the position and structural cross-section of the composite metal screw of the sandwich structure, **(B)** the top view of the structure and **(C)** the comparison diagram of locked plating (LP), far cortical locking (FCL), and sandwich locking (SWL) constructs: the proximal and distal ends of the LP are all locked, and the FCL screws are staggered by 9°; only the distal end is locked, and the proximal end is enlarged. The SWL screws are arranged in a straight line; only the distal end is locked and the proximal hole is not enlarged.

In order to better conduct biomechanical comparative analysis and stiffness verification of internal fixation constructs, three numerical models were established in Abaqus/CAE 2018 (Dassault Systems, Velizy-Villacoublay, France) based on previous work (Deng et al., 2021): the traditional locked plating constructs, the currently adopted FCL constructs, and the sandwich locking (SWL) constructs (Figure 2).

#### 2.1.1 Geometrical modelling and material properties

##### 2.1.1.1 Locked plating constructs and FCL constructs

A standard femoral cross-sectional bone model with a gap of 10 mm was established based on the fracture healing model for comparative analysis of internal fixation constructs. Implants were evaluated in normal femoral diaphysis surrogates to minimize inter-specimen variability (Bottlang et al., 2009; Bottlang et al., 2010b). We adopted cylindrical bone surrogates with a length of 200 mm, a diameter of 27 mm, and a wall thickness of 7 mm, modeled as linear elastic material (E = 17 GPa, ν = 0.3). The locking plate was 117 mm long, 17 mm wide, and 5.6 mm thick, and had a longitudinal curvature with a 750-mm radius. Self-tapping locking screws contained a shaft with a diameter of 3.2 mm in both locked plating and FCL constructs. Six threaded screw holes (6 mm diameter) were arranged in a staggered pattern in the FCL constructs (Figure 2C). The FCL screws for unicortical fixation in the far cortex consisted of a smooth screw midshaft with a diameter of 3.2 mm to bypass the near cortex, allowing for the elastic cantilever bending of the screw midshaft within a controlled motion envelope in the near cortex (Bottlang et al., 2009; Bottlang et al., 2010a). The plates and screws were manufactured with surgical grade titanium alloy (Ti-6Al-4V), which had a Young’s Modulus of 114 GPa and Poisson’s Ratio of 0.3.

##### 2.1.1.2 SWL constructs

Two modifications were made to the currently adopted FCL constructs; all other parameters remained the same. First, the 9° staggered arrangement of screws was omitted; the second modification was to optimize the topography of the screw structure, and to design grooves where the screw was close to the near cortex. Six threaded screw-holes (6 mm diameter) were arranged in a colinear pattern (Figure 2B). The structural differences between the three constructs are shown in Figure 2C.

The cross-section of the SWL screw and the optimization variables are shown in Figure 1B. Self-tapping composite metal locking screws had a 3.2 mm diameter shaft, and were only fixed in the far cortex. The screw shaft had a 10-mm long groove close to the near cortex to bypass it, allowing for elastic cantilever bending of the unicortical screw midshaft within a controlled motion envelope in the near cortex. There was a groove close to the near cortex to change the contact form of constructs in the sandwich composite metal screw. The Ti-6Al-4V was the skin of the sandwich composite, and the thickness (t) of the skin can be optimized by the optimization method. The properties of the core material can be optimized to reduce the stiffness of the construct, including Young’s Modulus (E) and Poisson’s Ratio (γ).

#### 2.1.2 Boundary and loading conditions

Model establishment was based on axial compression tests through a proximal sphere (rigid clamp), replicating the axial loading scenario of the bench-top test (Bottlang et al., 2009; Bottlang et al., 2010b). The distal ends of the bone models were fully constrained as boundary condition. Torsion was applied around the diaphyseal shaft axis (Supplementary Figure S8). In LP constructs, screws were assumed fully bonded to the bone and the plate using the tie constraint. In FCL and SWL constructs, screws were bonded to the far cortical bone and the plate using tie constraint. Relative motion in models have been considered for friction between the screws and near cortical bone. A standard Coulomb friction coefficient of 0.3 was employed based on some of the recent studies (Eser et al., 2010; MacLeod et al., 2012). For the static loading simulations, construct stiffness in non-osteoporotic bone surrogates was assessed under axial compression and torsion by loading to 1 kN and 10 Nm, respectively. In addition to the actuator displacement (displacement of the center of mass of the proximal sphere), interfragmentary motion under axial compression was recorded at the near and far cortices (Figure 2A).

The finite element model of each construct in this study was calculated and analyzed by using the structural mesh C3D8R and the mesh independence was further discussed in the Supplementary material (Supplementary Tables S3,S4).

### 2.2 Sandwich structural optimization

#### 2.2.1 Optimization objective

In our optimization, there were three intuitive optimal objects: controllable two-phase stiffness, nearly parallel interfragmentary motion and comprehensive strength.

First, the SWL constructs should reduce the stiffness of a standard locked plating construct by over 50%, and the stiffness of the normal model was 2.9 kN/mm in locked plating constructs, shown as:

$$K_{SWL}/K_{LP}\leq 50\%$$ (1)

Where $K_{SWL}$ represents the stiffness of SWL constructs and $K_{LP}$ represents the stiffness of locked plating constructs.

Second, these should induce nearly parallel motion at the near cortex and far cortex, and the difference of the displacement between the near cortex and far cortex should less than 15%:

$$2∙\frac{\delta_{A}-\delta_{B}}{\delta_{A}+\delta_{B}}<15\%$$ (2)

Where $\delta_{A}$ represents the displacement of the near cortex at the fracture ends, and $\delta_{B}$ represents the displacement of the far cortex at the fracture ends.

Third, the strength of the SWL constructs should meet the requirement of the safety assessment based on previous work (Deng et al., 2021). The result of optimization through the safety assessment represented the new constructs. There are two main aspects of the safety assessment: structural strength analysis based on the allowable stress, and high-cycle fatigue numerical analysis (further illustrated in the Supplementary material).

#### 2.2.2 Design method

The flowchart of the optimization method is shown in Figure 1C. In the optimization model, there were four optimization variables, including the two material parameters (Young’s Modulus (E) and the Poisson’s Ratio (γ) ) and two geometry parameters (the width of the groove (d) and the thickness of the titanium alloy layer (t)). However, there is not much difference between Poisson’s ratio (γ) of different materials, so the small change of Poisson’s ratio is not considered in the optimization process ( γ is constant, γ=0.3 ).

There are three steps in the optimization process. First, a random array was established for the optimization parameters ( E,γ,t, d ) using the Latin Hypercube method (Supplementary Table S1), and the value of the optimization objective ( K,δ, σ ) was obtained based on the finite element method. In the second step, the above results were used to establish the response relationship between the optimization parameters and the optimization target through the RSM method. Third, took the extremum of the von Mises stress ( σ ), axial stiffness ( K ) and displacement ( δ ) from the response surface relationship to get the optimized results. The value range of optimization variables are shown in Table 1. The three optimization variables were the input of the finite element model, and the algorithm applied in the optimization analysis was further illustrated in Supplementary Figure S1 in the Supplementary material (Zhou et al., 2013; López et al., 2017; Shang et al., 2019). The impact of the three optimization parameters on the optimal object was analyzed using the response surface model (RSM) method (Li et al., 2016; Patel and Gohel 2018; Li et al., 2019; Zhao et al., 2020).

**TABLE 1:** Parameter range for the optimization of a sandwich composite metal locking screw.

	Minimum value	Maximum value	Step size
Width of the groove (d) (mm)	0	2	0.20
Thickness of titanium alloy layer (t) (mm)	0.2	1	0.25
Young’s Modulus (E) (GPa)	80	262	10

### 2.3 Experiment validation

The established numerical simulation models were verified by statics tests and high-cycle fatigue tests, and the fatigue damage of each construct was explored through defect detection. The internal fixation system used in the mechanical experiments in this study was consistent with the geometric parameters used in the numerical model. Implants were custom manufactured by a company specializing in the production of orthopedic implants (Geasure, Changzhou, Jiangsu). Implants were evaluated in surrogate specimens of the femoral diaphysis to minimize inter-specimen variability (as further illustrated in Supplementary Figure S2 in the Supplementary material).

Axial compression testing was performed on the three constructs (n = 5 in each group) using a biaxial universal material testing system (Instron e10000, Instron, Massachusetts, United States) (Supplementary Figure S3). For the static loading tests, an axial compression load was applied under load control with an increment of 100 N, up to 1000 N. We performed three repeated case loadings for each sample, gave each sample at least 12 min of recovery time before each load repetition, and recorded displacements and loads throughout the loading process. The structural axial stiffness of the sample was calculated from the displacement-load data, and each curve area was segmented and the slope calculated. The slope of a group of samples was the average of the slopes obtained from three repeated loads. Further, we performed high-cycle dynamic fatigue tests of 1,000,000 cycles (waveform: sine wave) at a rate of 5 Hz according to the load levels presented in Supplementary Figure S4 in the Supplementary material.

All samples were carefully collected, sorted, and cleaned, and then micro-nano tomography was performed to observe the damage location and damage mode of the samples under high cycle fatigue testing (further illustrated Supplementary Table S2 and Supplementary Figure S5 in the Supplementary material).

### 2.4 Outcome evaluation

To evaluate the stability and safety of the optimized and redesigned sandwich composite metal locking screws, we compared the differences in biomechanical behavior between screws in three constructs for both loading mode, including 1) construct stiffness of the fixation models for each load, and 2) the interfragmentary motion results at the near and far cortices. Axial stiffness was calculated by dividing the axial load amplitude by the actuator displacement amplitude. Torsional stiffness was calculated by dividing the torsion amplitude by the amplitude of rotation (α) around the diaphyseal axis. Torsional stiffness was multiplied by the unsupported specimen length to derive torsional rigidity. What’s more, we compared the differences in biomechanical safety in three constructs for both loading mode, included 1) the von Mises stress distribution and peak values of the screws and bone models, 2) the average von Mises stress of all elements of the screws and bone models, and 3) numerical and experimental results of high-cycle fatigue test.

For statistical analysis, the average von Mises stress of all elements of the screws and bone models and the construct stiffness were compared among three groups individually, for both loading mode using one-way ANOVAs with Bonferroni post-hoc tests. For all statistical analyses, a level of significance of *α* = 0.05 were used to detect significant differences.

## 3 Results

### 3.1 Optimal design results

**FIGURE 3:**
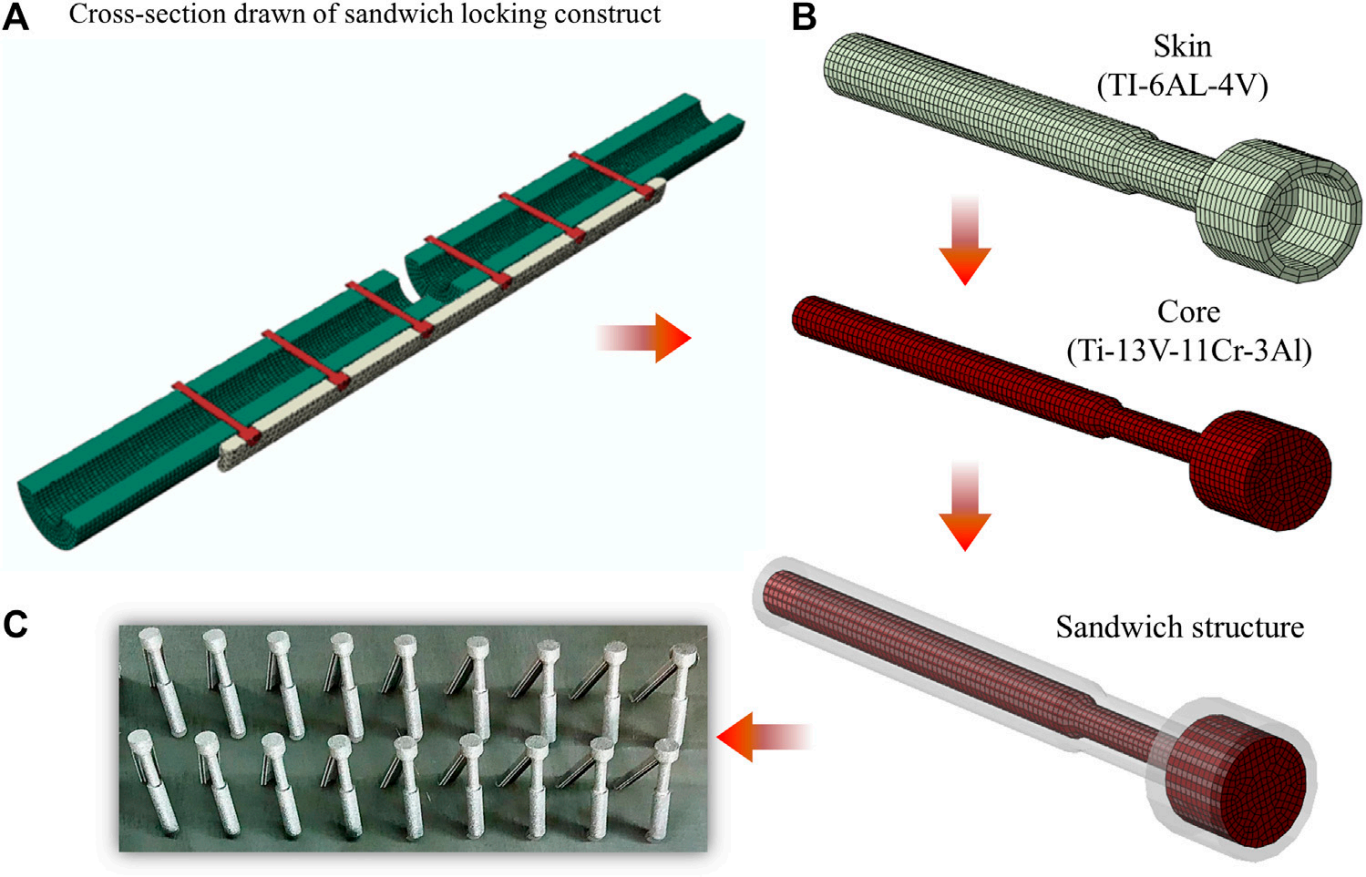
Structure schematic diagram of the optimized finite element model of the SWL screw. **(A)** Cross-section drawn of sandwich locking construct. **(B)** The best result of optimization is d = 0.25 mm, t = 0.65 mm and E = 98 GPa. Considering the influence of material compatibility, titanium alloy (Ti-13V-11Cr-3Al) was selected as the core material (Young’s modulus E = 98 GPa and Poisson’s ratio γ = 0.3). **(C)** Implant manufactured by 3D printing using titanium alloy (TI-6AL-4V) as a proof-of-concept.

Using the optimization method, the sandwich composite metal locking screw was designed for controllable two-phase stiffness, near parallel interfragmentary motion, and comprehensive safety. The final results of the three variables are shown below. The width of the groove (d) was 0.25 mm, and the thickness of the titanium alloy layer (t) was 0.65 mm. The Young’s Modulus (E) was 98 GPa, so titanium alloy (Ti-13V-11Cr-3Al) was chosen as the core material (Young’s Modulus, E=98 GPa ; Poisson’s Ratio, γ=0.3 ). The optimized sandwich composite metal screw is shown in Figure 3. As a preliminary proof-of-concept, the screws had been additively built with a metal 3D printer Renishaw AM 400 (Renishaw plc, United Kingdom) (further illustrated in the Supplementary Material).

### 3.2 The construct stiffness and displacements

The new SWL constructs have the same force-contact pattern as the FCL constructs. Table 2 summarized the construct stiffness in axial compression and torsion, and the simulation results have been verified by comparison with our experimental results and those reported in Bottlang et al. (2014). It can be seen that both the simulation results and the experimental results were highly consistent with the classical FCL construct biomechanical experimental results.

**TABLE 2:** The stiffness results of each group of experiments under axial compression (0–1000 N) and torsion (0–10 Nm) loading conditions.

		Locked plating	Sandwich locking^a^	Far cortical locking^a^	*p* value^‡^
Axial stiffness (kN/mm)	Bottlang et al. (2009)	2.9 ± 0.13	——	0.36 ± 0.05/2.26 ± 0.08	<0.001/<0.001
	Our simulation	2.92	0.47/3.06	0.67/2.86	——
	Our experiment	2.90 ± 0.25	0.66 ± 0.04/3.09 ± 0.15	0.84 ± 0.19/2.19 ± 0.12	<0.001/<0.001
Torsional rigidity (Nm^2^/deg)	Bottlang et al. (2009)	0.4 ± 0.03	——	0.17 ± 0.04/0.32 ± 0.01	<0.001/<0.001
	Our simulation	0.38	0.16/0.27	0.17/0.31	——

a The stiffness data are given as the initial value followed by the secondary value. ‡The first *p* value pertains to the comparison among the initial FCL, value, initial SWL, value and the locked plating value, and the second *p* value pertains to the comparison among the secondary FCL, value, secondary SWL, value and the locked plating value.

**FIGURE 4:**
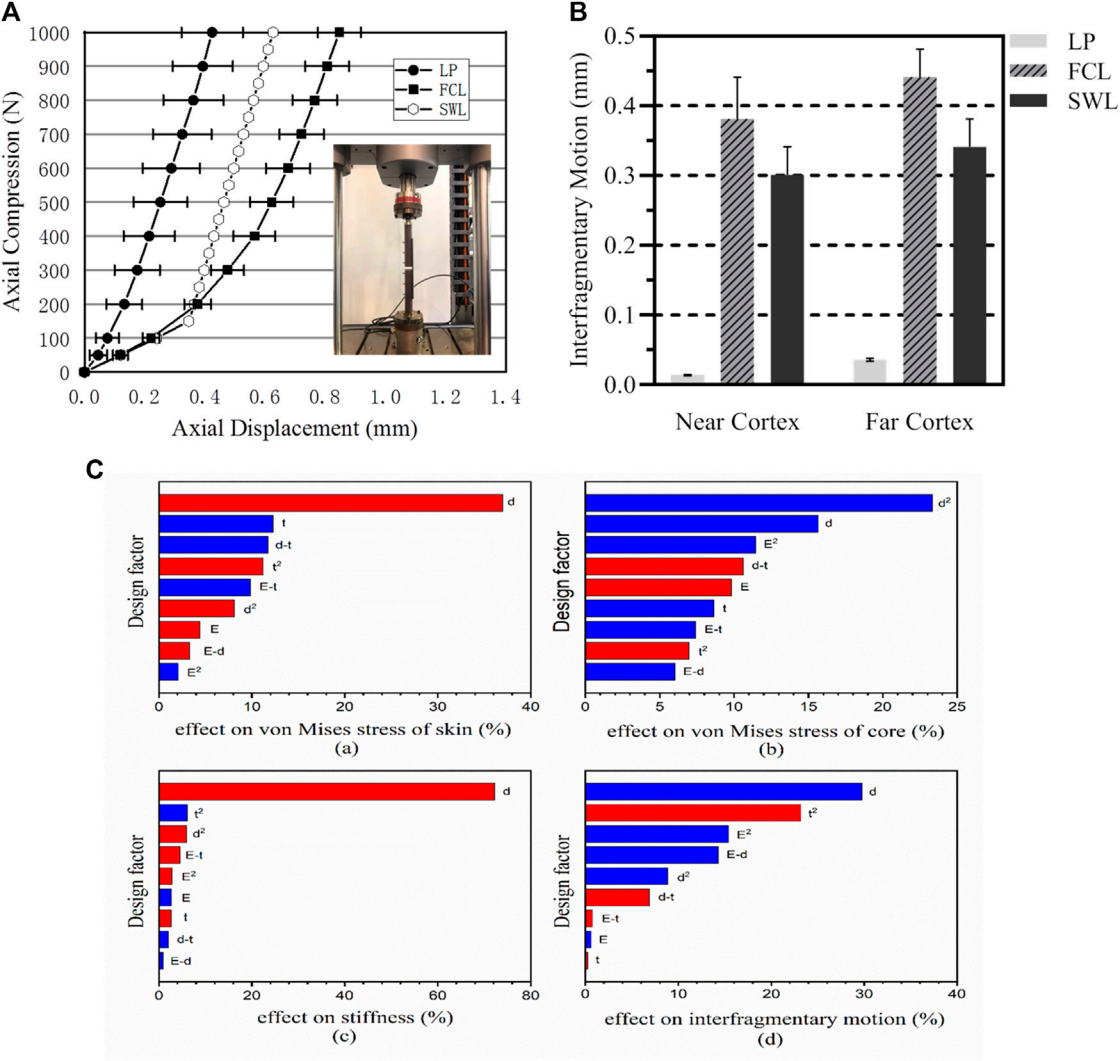
Structural displacement analysis. **(A)** Comparison of the stiffness of the three structures in the non-osteoporotic model in axial compression. **(B)** At 150 N of loading, the initial stiffness of three constructs induced comparable amounts of interfragmentary motion at the near and far cortex. LP: Locked plating constructs; FCL: Far cortical locking constructs; SWL: Sandwich locking constructs. **(C)** Factor sensitivity analysis of each design parameter.

Under an axial load of 150 N, the axial compressive stiffness of the SWL construct was 0.47 kN/mm , which was 83.9% lower than that for the locked plating construct (2.92 kN/mm ). For axial loads greater than 150 N, the SWL construct second-phase stiffness was 3.06 kN/mm and maintained a strong stiffness. Axial loads above 150 N caused the sandwich composite screws to come into contact with the proximal cortical bone, which provided additional structural support, thereby increasing the secondary stiffness. In torsion, the initial torsional stiffness of the SWL construct was 64% lower than that of the locked plating construct (0.16 Nm^2^/deg versus 0.38 Nm^2^/deg). For torsion with torque greater than 2 Nm, the secondary stiffness of the SWL structure increased to 0.276 Nm^2^/deg, which was still 13% lower than that of the FCL construct. The stiffness differences of the three constructs are shown in Figure 4A.

The groove of the sandwich composite screw changes the contact pattern between the screw and the cortical bone, so that the displacement difference between the proximal and distal ends of the fracture was small, and an approximately parallel pattern was achieved. Figure 4B showed the difference in displacement between the proximal and distal ends of cortical bone for the three constructs at 150 N. Within the initial stiffness range of the SWL construct, an axial load of 150 N induced almost parallel displacements at the fracture site, with similar displacement magnitudes at the proximal (0.301 ± 0.04 mm) and distal (0.341 ± 0.04 mm) ends of the cortical bone. The displacement difference between the proximal and distal ends of the cortical bone in the SWL construct was 0.040 mm, which was smaller than that of the FCL construct (0.059 mm). In the locked plating construct, the corresponding displacement in the proximal cortex (0.02 ± 0.01 mm) was significantly smaller than that in the distal cortex (0.05 ± 0.02 mm) (*p* < 0.01).

### 3.3 The safety assessment results

**FIGURE 5:**
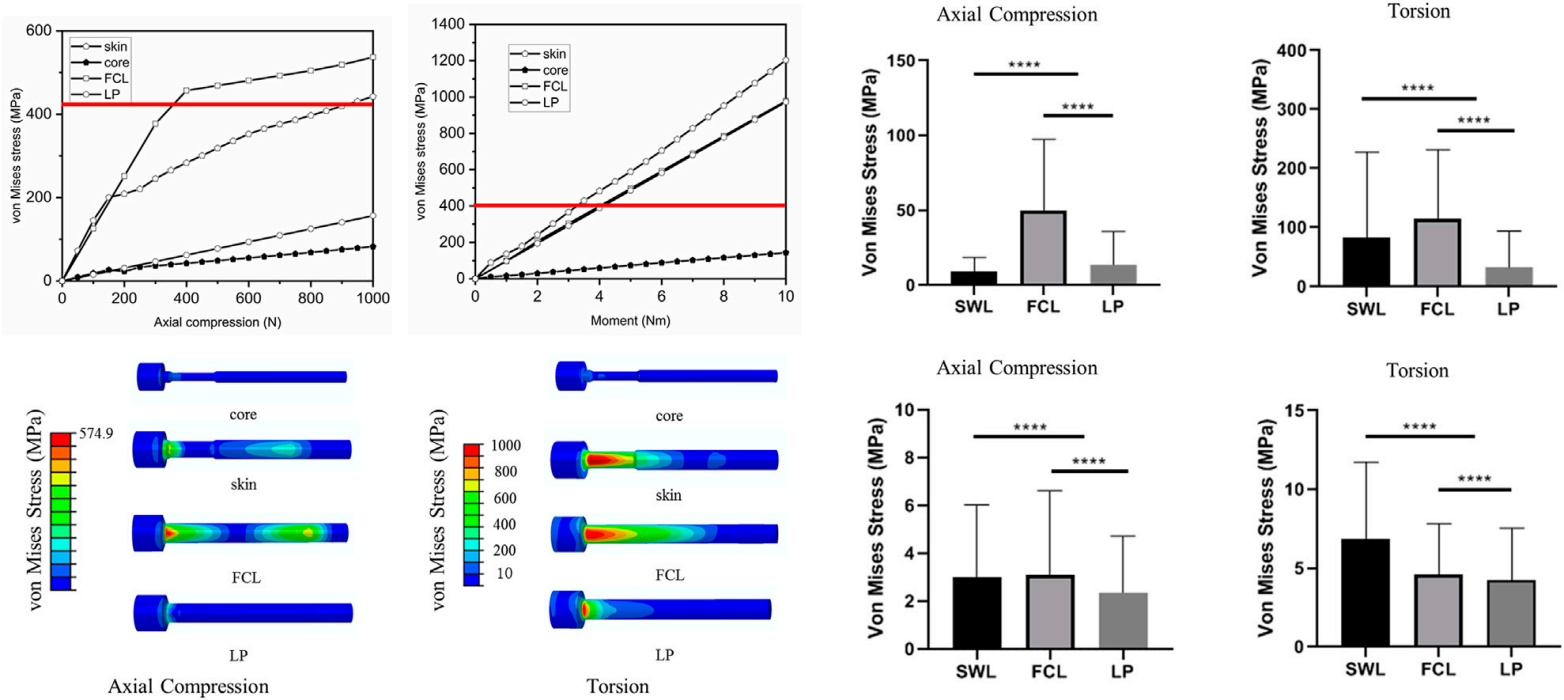
The von Mises stress results for the three constructs. **(A,B)** Under axial compression and torsion load, the maximum von Mises stress of each screw varies with load and the allowable stress of titanium alloy (red line). **(C,D)** Maximum von Mises stress cloud diagram of each screw under 1000 N axial compression and under 10 Nm. **(E–H)** The average von Mises stress of all elements of the screws **(E,F)** and bone models **(G,H)** were compared among three groups individually for each loading mode. **** means *p* < 0.0001.

As the factor of safety (n) was 2, the allowable stress can be calculated by $\left[\sigma \right]=\frac{\sigma_{Y}}{n}=\frac{\sigma_{Y}}{2}$ . The allowable stresses of the two titanium alloys were ${\left[\sigma \right]}_{Ti-6Al-4V}=412.5MPa$ and ${\left[\sigma \right]}_{Ti-13V-11Cr-3Al}=415MPa$ , respectively. The maximum value of the von Mises stress is less than the allowable stress to ensure structural safety. We evaluated the fracture risk of the screws that had the maximum deformation among the screws in each model. The maximum von Mises stresses of the screws were shown in axial compression loading and torsion, in comparison with the allowable stress (red line) (Figures 5A,B). Under the axial compressive load of 400 N, the maximum von Mises stress of the outer layer metal titanium and the core structure alloy is 300.7 and 45.8 MPa, respectively, which were all less than the allowable stress of the material. The maximum von Mises stress of the FCL screws exceeded the allowable stress of the titanium alloy (574.9 MPa), which was 1.912 times the maximum von Mises stress of the SWL screws, so the risk of screw breakage and secondary fracture of the SWL construct was lower. For the torsional condition, under a physiological torsional load of 2 Nm, although the SWL constructs did not use a 9° staggered arrangement of screw placement, the maximum von Mises stress of the SWL was lower than the allowable stress. The maximum von Mises stress distributions of the three structural screws are shown in Figures 5C,D **,** and the von Mises stress of SWL screws was generally lower than that of the FCL screws under axial compressive load. When the axial compressive load was below 800 N or the torsion was below 3 Nm, the maximum von Mises stress of SWL screws was lower than the allowable stress. In addition, the average von Mises stresses of all elements of the screws and bone models were compared among the three groups individually for each loading mode (Figures 5E–H). At 1000 N axial compression, the average von Mises stress of all elements of the SWL construct was significantly smaller than that of the FCL constructs in both screw and bone models (*p* < 0.0001). At 10 Nm torsion, the average von Mises stress of the screw elements of the SWL construct was significantly smaller than that of the FCL construct (*p* < 0.0001). In this study, the stresses of plates in three constructs were in the safe range, far lower than the allowable stresses (Supplementary Tables S5, S6).

**FIGURE 6:**
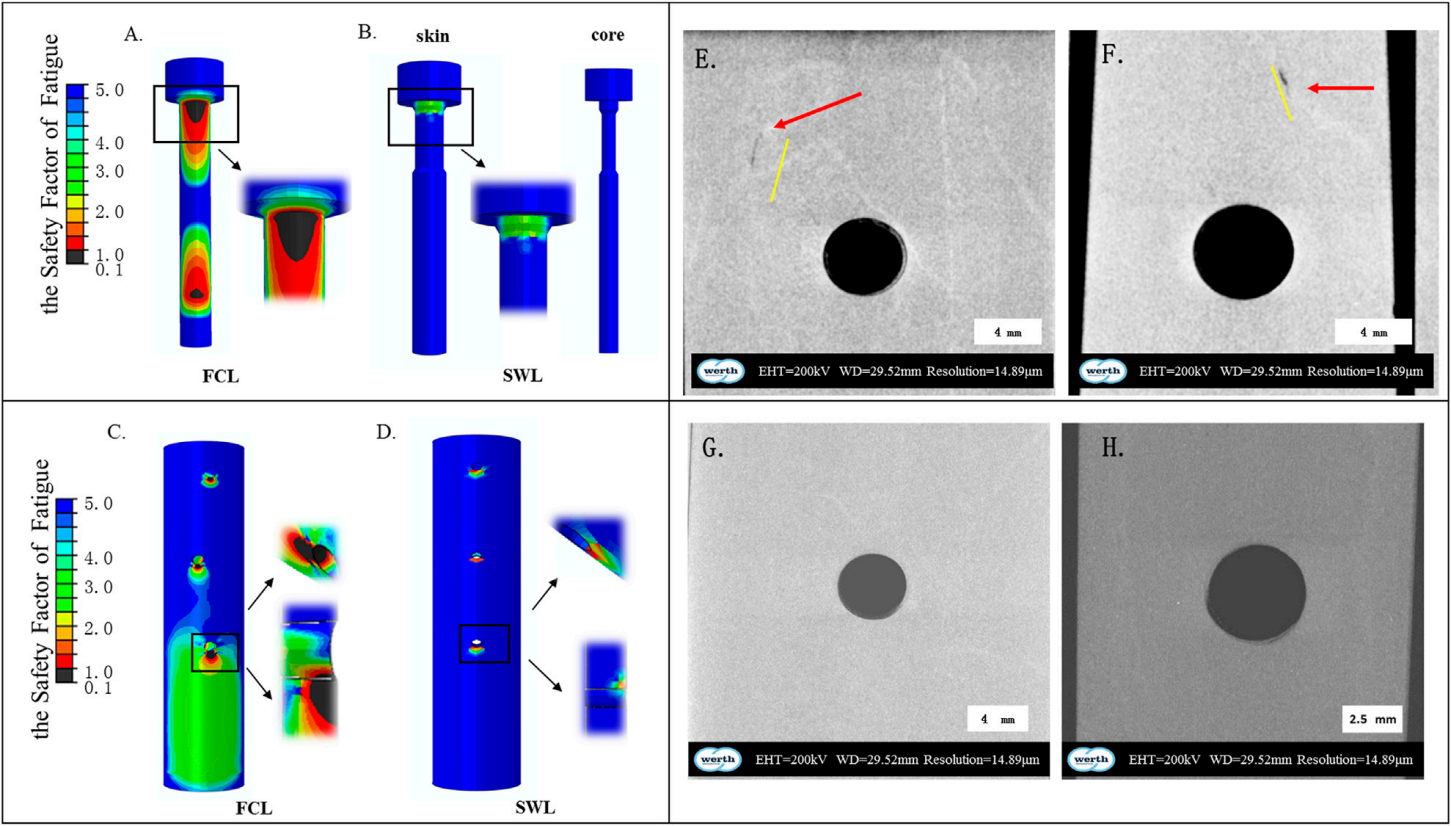
Numerical simulation and experimental results of high-cycle fatigue of three constructs. **(A–D)** High-cycle fatigue assessment and analysis based on FE-safe, the distribution cloud map of fatigue safety factors. **(E–H)** The appearance of crack initiation in the area of screw-bone interface at the far cortices: FCL construct samples **(E,F)**, LP construct samples **(G)**, SWL construct samples **(H)**.

For the fatigue life analysis results shown in Figure 6, the area with a fatigue safety factor of one or more are the safe area of the construct, and the red part is the dangerous area, showing the area with a safety factor below one. The numerical simulation results indicated the level of fracture risk. The minimum fatigue safety factor for SWL constructs exceeded 1.7. Fatigue safety factors and fatigue life are summarized in Table 3. Compared with the FCL construct (0.2), the SWL construct increased the safety factor to 5. The fatigue life of the sandwich screws exceeded 1 million cycle loads, and the fatigue life of the bone in the SWL constructs was also greater than that in the FCL constructs.

**TABLE 3:** Three types of structural minimum safety factors and fatigue life prediction results.

	Far cortical locking		Locked plating		Sandwich locking		
	Bone	Screw	Bone	Screw	Bone	Screw (skin)	Screw (core)
The minimum safety Factor of fatigue (m)	0.650	0.2	2.594	5	4.731	1.7	5
Fatigue life × 1,000, 000 cycle	0.634	0.636	≫ 1	≫ 1	0.744	≫ 1	≫ 1

The micro-nano tomography results were shown in Figures 6E–H, and the mechanical experimental results were in good agreement with the fatigue numerical prediction results. The results showed that all samples from the FCL group had the same damage pattern, that is, the greatest structural damage was produced in the area of the junction of the distal cortical bone screw and the artificial bone. Figures 6E,F shows the appearance of crack initiation in the area of the screw-bone interface at the far cortices. The red arrow indicated the location of structural damage under high-cycle fatigue, and the yellow line indicated the crack morphology of the structural damage. It can be seen that the FCL constructs had cracks or defects in the non-osteoporotic bone model, while no cracks or defects were detected in the locked plating constructs and SWL constructs (Figures 6G,H). Therefore, we have reason to believe that the safety of the SWL contructs meets our design requirements.

### 3.4 Design parameter sensitivity analysis

The influence of the three design parameters on the optimal objects during the optimization process was calculated. Through regularized responses on factors, it can be seen that the square term has a considerable effect on the result (over 45%). Figure 4C shows the sensitivity analysis results calculated by the RSM method, and the R-squared value was 0.9563. The red represents positive correlation and the blue negative correlations. It is evident that the width of the groove (d) had a positive effect on the von Mises stress of the outer titanium alloy, while d^2^ had a negative effect on the core structure.

## 4 Discussion

This study provided a design method for the sandwich composite metal locking screw, and optimized a new construct with controllable two-phase stiffness, near parallel interfragmentary motion, and high fixation construct safety to promote secondary fracture healing. With the development of sandwich structure 3D print technology (Sypeck and Wadley 2002; Sarvestani et al., 2018), we can reasonably assume that the sandwich composite metal screw is an ideal choice for dynamic fixation of fractures.

The sandwich screw is a cantilever beam with different working lengths under increasing load. The groove ensured the bone as a rigid body under a longer cantilever length, until the screw contacted with the near cortex. The larger is the width of the groove, the greater is the contact load. The above theory concurred with the sensitivity analysis shown in Figure 4C. The elasticity modulus of the core material and the depth of the groove influenced the two stiffnesses of the new constructs. Of course, the core material was mainly responsible for ensuring the safety of the construct. In considering biocompatibility and enhanced corrosion resistance, the Ti-6Al-4V was adopted as the protective skin of the sandwich screw.

In axial compression, the initial stiffness of the far cortical locking construct was 52% lower than that of the locked plating construct (Figure 4A). Secondary bone healing requires flexible fixation and relative stability to enable interfragmentary motion to stimulate callus formation (Perren 1979; Claes et al., 1998; Duda et al., 2002; Hente et al., 2004; Zhang et al., 2012; Benoit et al., 2016). The new constructs exhibited a biphasic stiffness profile with an initial stiffness and a secondary stiffness similar to the FCL construct (Bottlang et al., 2009; Deng et al., 2021). Studies demonstrated that asymmetric gap closure with locking plates caused asymmetric callus formation, with callus formation decreasing from the far cortex towards the near cortex (Bottlang et al., 2010a; Lujan et al., 2010). Clinically, the nearly parallel interfragmentary motion provided by FCL constructs should contribute to symmetric callus formation across the entire fracture site (Bottlang et al., 2014; Adams et al., 2015; Moazen et al., 2016; Kidiyoor et al., 2019). The new constructs with sandwich screws had similar parallel interfragmentary motion (Figure 4B), which may have similar symmetric callus formation. With similar biomechanical theory for FCL constructs, the new construct can theoretically promote secondary healing of fractures.

To ensure the safety of the new constructs, it is necessary to choose allowable stresses and restrict the applied load to a lower value than the construct can fully support (Casavola et al., 2011; Badalassi et al., 2014; Deng et al., 2021). Considering the inescapable microcracks of the implant and crack growth, allowable stress is a powerful instrument for assessing the implant in terms of strength theory and suitability of the metal material (Gross and Abel 2001; Sarma and Adeli 2005; Manral et al., 2020). Considering defects in the implant material and the influence of processing technology, greater design load and safe allowable stress should be adopted in the biomechanical assessment of screw failure risk (Agius et al., 2018; Anitua et al., 2018; Rahimizadeh et al., 2018; Choi et al., 2019). The fracture fixation constructs should provide sufficient biomechanical safety, particularly in terms of avoiding fatigue damage during 3–4 months of rehabilitation training. From the simulation results (Figures 6A–D), the safety of the new construct was guaranteed. From the experimental results, it can be seen that under the same conditions, the new construct had better fatigue resistance (Figures 6E–H). The high cycle fatigue numerical analysis results of this study indicated that both the bone models and the screws of FCL construct were at risk of fatigue failure under 1,000,000 cycles of cyclic loading, and fatigue life prediction analysis indicated that the fatigue failure of the bone models will occur first (Table 3). After micro-nano tomography and defect analysis of the bone models, fatigue damage was indeed found in the bone models of FCL constructs, which was consistent with the results of our fatigue numerical analysis. However, the results indicated that there was no fatigue damage of any FCL screws. It may be that the fatigue damage of the bone models released the stress on the screw, so that the screws did not suffer fatigue damage after the bone model is damaged as in numerical analysis shown. In addition, in current FCL constructs, the screws need to be arranged in a 9° staggered arrangement to ensure the torsional stiffness of the constructs, but this arrangement increases the difficulty of the surgery. Due to the design concept of the FCL constructs, to ensure that the stiffness of the first phase is small and the load when it is converted to the stiffness of the second phase is large, it is necessary to create a larger movable groove at the proximal cortical bone, which increases the complexity of the operation. The 9° staggered arrangement of the screws also means that greater trauma is added to the original fracture, further disrupting the continuity of the cortical bone. The surgical fixation plan must have universality for the various fractures of each patient with less damage to the bone.

The limitations of this study are as follows: In the model verification experiment, because at present the current technology cannot realize the grafting and 3D printing of 0.65-mm Ti-6Al-4V on titanium alloy (Ti-13V-11Cr-3Al), we only used the SWL screws made of core material titanium alloy (Ti-13V-11Cr-3Al). However, the SWL model in this study is in an ideal condition that Ti-6A1-4V can fully bond with Ti-13V-11Cr-3Al. This combined structure relies on the manufacturing level and needs more fabricating cost. The current metal 3D printing technology is still difficult to achieve fine grafting and printing on complex 3D structures. Thus, the use of this new structure depends on the development of the manufacturing process. In addition, this study was based on an ideal fracture model on cylindrical bone models and did not represent the various complex situations of actual clinical fractures (such as more complex fracture lines and force lines).

In conclusion, in this study, the finite numerical simulation calculation was used to intelligently optimize the locking screws of the FCL construct under multiple working conditions. The SWL construct theoretically maintains its biomechanical safety and fatigue resistance while maintaining excellent mechanical properties for fracture internal fixation. The newly designed composite metal sandwich locking screw can theoretically achieve elastic fixation, progressive stiffness, uniform load distribution, and parallel interfragmentary motion of the fracture end in the far cortical locking construct, while maintaining better fatigue resistance and torsion resistance to the internal fixation construct. Additional studies are required to assess SWL screws performance in combined loading modes and to determine if SWL constructs effectively promote secondary bone healing *in vivo*.

## Data Availability

The original contributions presented in the study are included in the article/Supplementary Material, further inquiries can be directed to the corresponding authors.
