# Supplementary material for: Optimal design and biomechanical analysis of sandwich composite metal locking screws for far cortical locking constructs
Source: Front Bioeng Biotechnol. 2022 Sep 27;10:967430. doi: 10.3389/fbioe.2022.967430 (PMC9551571; doi:10.3389/fbioe.2022.967430)
Supplement: Supplementary file 1 [file DataSheet1.DOCX]

**Supplementary information**

**1. Supplementary description of optimization algorithm**

Based on the Isight platform, the intelligent cycle optimization design was carried out (as shown in **Figure S1**), and the results of each cycle can be automatically extracted through programming scripts. As shown in the flow chart, the DOE1 module was the experimental design module, which was responsible for the recording of cyclic design parameters, can automatically update the position of parameter extraction and sampling, and output the corresponding optimization parameters and optimization results at the same time. This study used the Latin hypercube sampling method as an optimized experimental design technique. It is a kind of stratified random sampling, which can efficiently sample from the distribution interval of variables. Assuming that there are now k variables, and we now want to take N samples from their specified interval, the cumulative distribution of each variable is divided into the same In N small intervals, a value is randomly selected from each interval, and the N values of each variable are randomly combined with the values of other variables. Unlike random sampling, this method makes each marginal distribution stratified by maximizing, to ensure full coverage of each variable range. The most important characteristic of Latin hypercube sampling is that it can generate sampling samples of any size. Abaqus module: restart the design cycle according to the input data and extract the optimization indicators such as structural displacement, interfragamentary motion and allowable stress of a new round of optimization scheme. Optimization module: Perform structural optimization and composite material selection of screws according to the optimization design objectives in the above methods. Calculator module: Convert structural node data into optimization target parameter results. Fe-safe module: perform fatigue safety assessment of the constructs, optimization schemes with a fatigue safety factor greater than a predetermined value will be rejected, and the design cycle will be restarted.


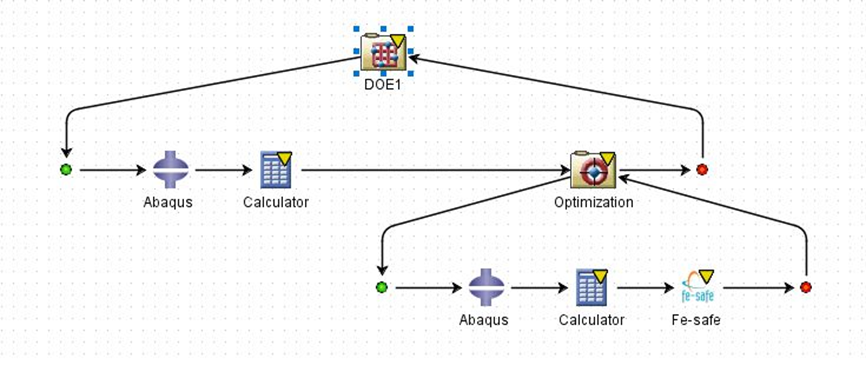


**Figure S1 Flow chart for optimization of intelligent loop design structure built by Isight Platform.**

The optimization method mainly includes two aspects: one is the experimental design based on the Latin hypercube sampling method, and the other is the optimization solution based on the outer point penalty function method. The Latin hypercube sampling method is a multidimensional stratified sampling method, which firstly divides the design space evenly, divides all the control variables of the study equally, and then determines n points by free combination and each factor is only considered once, finally make more combinations for each point and each factor. The Latin hypercube sampling method can generate sampling samples of any size. It can significantly improve the global optimization ability and optimization efficiency of the dynamic surrogate model optimization design method, and is suitable for the field of modern engineering design optimization using high-precision analysis models. In this study, 80 samples were selected by the Latin hypercube method for experimental design, and the combination of the 80 samples is shown in **Table S1**. Based on the optimal solution obtained by the experimental design, the exterior penalty function method was used to optimize the solution, whichwas widely used in the research of constrained optimization problems. Under the condition of using boundary value constraints, this method can efficiently find the target value required by the algorithm. The exterior penalty function method can bring the value of the penalty function to an infinite value, so that the design variables in the infeasible region can be brought back to the feasible region, and the optimization design goal can be realized. In the optimization part, python script was used to perform Abaqus modeling and calculation, and the optimization target parameters such as stress, proximal and distal control point displacement, and construct stiffness are extracted. The mold process also includes the optimized thickness, depth, elastic modulus and other three parameters.

| Table S1 Latin hypercube sampling method sample data. | | | | | | | | | |
| --- | --- | --- | --- | --- | --- | --- | --- | --- | --- |
| Sequence sets # | | E | d | t | Sequence sets # | E | d | t |
| 1 | 83840 | | 1.924 | 0.767 | 41 | 174051.8 | 0.43 | 0.251 |
| 2 | 86093.2 | | 0.228 | 0.635 | 42 | 176305 | 0.582 | 0.686 |
| 3 | 88346.4 | | 0.962 | 0.322 | 43 | 178558.2 | 1.418 | 0.2 |
| 4 | 90610.08 | | 1.089 | 0.453 | 44 | 180811.4 | 1.646 | 0.413 |
| 5 | 92863.28 | | 0.987 | 0.787 | 45 | 183064.6 | 0.557 | 1 |
| 6 | 95116.48 | | 1.19 | 0.271 | 46 | 185328.3 | 0.203 | 0.433 |
| 7 | 97369.68 | | 1.519 | 0.605 | 47 | 187581.5 | 1.747 | 0.494 |
| 8 | 99622.88 | | 1.215 | 0.281 | 48 | 189834.7 | 0.734 | 0.757 |
| 9 | 101886.6 | | 1.114 | 0.514 | 49 | 192087.9 | 1.772 | 0.463 |
| 10 | 104139.8 | | 0.38 | 0.311 | 50 | 194341.1 | 1.975 | 0.352 |
| 11 | 106393 | | 2 | 0.585 | 51 | 196594.3 | 0.101 | 0.818 |
| 12 | 108646.2 | | 0.608 | 0.473 | 52 | 198858 | 0.684 | 0.524 |
| 13 | 110899.4 | | 0.481 | 0.909 | 53 | 201111.2 | 1.291 | 0.291 |
| 14 | 113152.6 | | 0.354 | 0.808 | 54 | 203364.4 | 0.785 | 0.797 |
| 15 | 115416.2 | | 0.937 | 0.666 | 55 | 205617.6 | 1.544 | 0.382 |
| 16 | 117669.4 | | 1.266 | 0.22 | 56 | 207870.8 | 1.139 | 0.848 |
| 17 | 119922.6 | | 1.165 | 0.868 | 57 | 210134.5 | 0.506 | 0.362 |
| 18 | 122175.8 | | 1.342 | 0.595 | 58 | 212387.7 | 1.823 | 0.504 |
| 19 | 124429 | | 0 | 0.615 | 59 | 214640.9 | 1.392 | 0.544 |
| 20 | 126692.7 | | 0.658 | 0.737 | 60 | 216894.1 | 0.152 | 0.696 |
| 21 | 128945.9 | | 1.241 | 0.342 | 61 | 219147.3 | 0.759 | 0.403 |
| 22 | 131199.1 | | 0.709 | 0.423 | 62 | 221411 | 1.063 | 0.777 |
| 23 | 133452.3 | | 1.848 | 0.747 | 63 | 223664.2 | 0.051 | 0.889 |
| 24 | 135705.5 | | 0.025 | 0.554 | 64 | 225917.4 | 0.405 | 0.919 |
| 25 | 137969.2 | | 1.367 | 0.372 | 65 | 228170.6 | 0.127 | 0.899 |
| 26 | 140222.4 | | 1.696 | 0.332 | 66 | 230423.8 | 0.861 | 0.565 |
| 27 | 142475.6 | | 1.57 | 0.878 | 67 | 232687.4 | 0.278 | 0.575 |
| 28 | 144728.8 | | 0.532 | 0.939 | 68 | 234940.6 | 0.177 | 0.443 |
| 29 | 146982 | | 1.038 | 0.646 | 69 | 237193.8 | 0.911 | 0.21 |
| 30 | 149245.7 | | 1.899 | 0.241 | 70 | 239447 | 1.595 | 0.656 |
| 31 | 151498.9 | | 0.076 | 0.534 | 71 | 241700.2 | 1.873 | 0.727 |
| 32 | 153752.1 | | 1.722 | 0.392 | 72 | 243953.4 | 1.949 | 0.484 |
| 33 | 156005.3 | | 0.456 | 0.98 | 73 | 246217.1 | 1.013 | 0.676 |
| 34 | 158258.5 | | 1.494 | 0.838 | 74 | 248470.3 | 1.468 | 0.828 |
| 35 | 160511.7 | | 0.886 | 0.99 | 75 | 250723.5 | 0.329 | 0.97 |
| 36 | 162775.4 | | 1.316 | 0.716 | 76 | 252976.7 | 0.81 | 0.23 |
| 37 | 165028.6 | | 0.633 | 0.949 | 77 | 255229.9 | 1.62 | 0.301 |
| 38 | 167281.8 | | 1.797 | 0.261 | 78 | 257493.6 | 0.304 | 0.959 |
| 39 | 169535 | | 1.443 | 0.706 | 79 | 259746.8 | 0.253 | 0.858 |
| 40 | 171788.2 | | 0.835 | 0.929 | 80 | 262000 | 1.671 | 0.625 |

**2. Supplementary description of model validation**

**2.1 Implants and specimens**

For biomechanical evaluation of internal fixation structures in non-osteoporotic bone, a 27-mm diameter was fabricated using the same materials and dimensions as the proven mid-sized fourth-generation composite sawbones femoral diaphysis, cylindrical bone substitute with 7 mm wall thickness (#3403, Pacific Research Laboratories, Vashon, Washington). Implants were custom manufactured by a company specializing in the production of orthopedic implants (Geasure, Changzhou, Jiangsu). All plates and screws except SWL screws were custom manufactured from medical surgical grade titanium alloy (Ti-6Al-4V). The SWL screws were are manufactured with an optimized core material titanium alloy (Ti-13V-11Cr-3Al). Because at present the current technology cannot realize the grafting and 3D printing of 0.65-mm Ti-6Al-4V on titanium alloy (Ti-13V-11Cr-3Al), we only used the SWL screws made of core material titanium alloy (Ti-13V-11Cr-3Al). However, based on the above sensitivity analysis results, it can be seen that the effect of the skin layer Ti-6Al-4V on the construct stiffness was minimal. We believe that the lack of Ti-6Al-4V will not have much impact on construct stiffness and fatigue resistance.

During surgery, the traditional assembly method can be used to drill holes in one line, as shown in Figure 2A. To simulate biological fixation with preservation of periosteal perfusion, spacers were used to keep a bone–plate distance of 2 mm. Used a 4 Nm torque-limiting screwdriver to tighten the screws, and placed three screws in the first, third and fifth holes of the steel plate, where the spacing between the screw holes is 36mm, and tighten three screws on one side. Tighten the three screws on the other side (As shown in **Figure S2**).


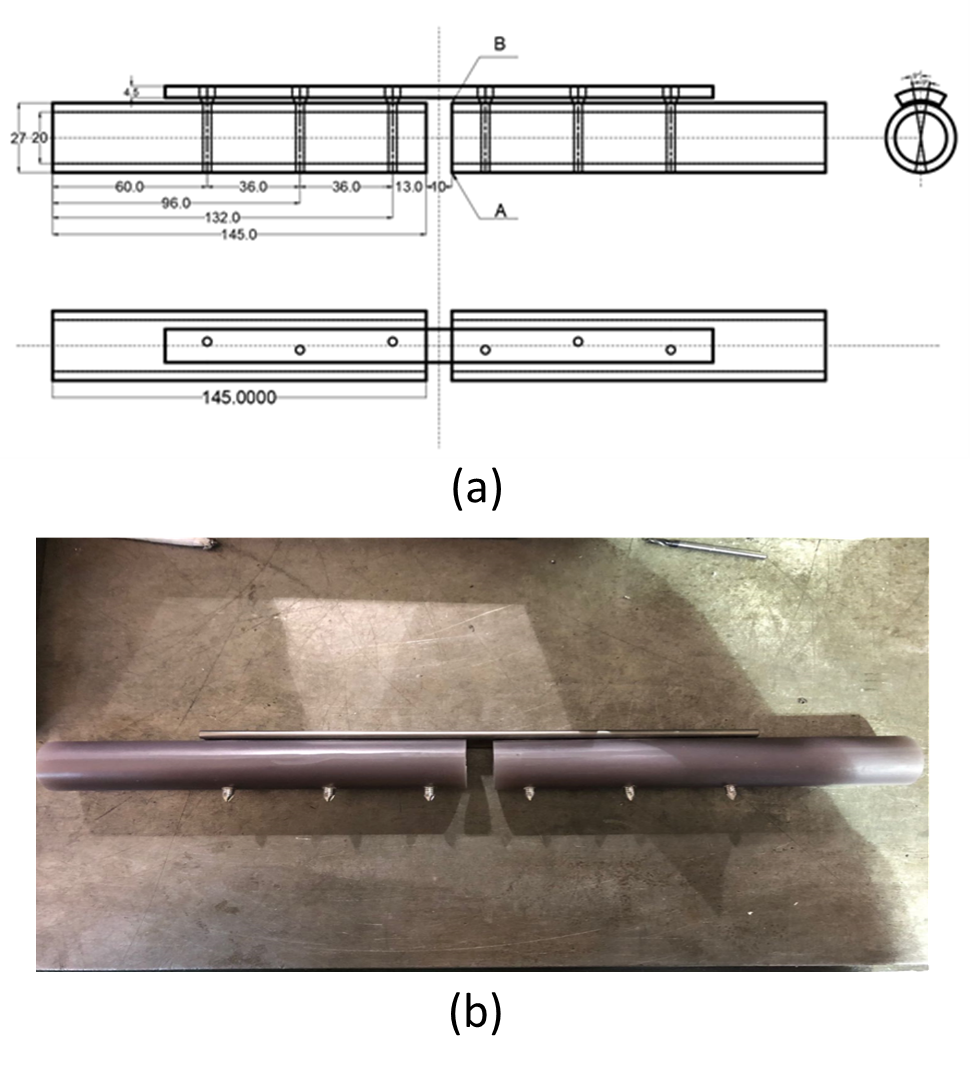


**Figure S2 Tooling diagram of the internal fixation system**

(a) Diagram of the tooling of the contralateral locking internal fixation system, (b) The assembled locking steel plate internal fixation system.

**2.1 Load condition settings**

Axial compression testing was performed on the three constructs (n=5 in each group) using a biaxial universal material testing system (Instron e10000, Instron, Massachusetts, USA) **(Figures S3).** For the static loading tests, an axial compression load was applied under load control with an increment of 100 N, up to 1000 N. We performed three repeated case loadings for each sample, gave each sample at least 12 min of recovery time before each load repetition, and recorded displacements and loads throughout the loading process. The structural axial stiffness of the sample was calculated from the displacement-load data, and each curve area was segmented and the slope calculated. The slope of a group of samples was the average of the slopes obtained from three repeated loads. Further, we performed high-cycle dynamic fatigue tests of 1,000,000 cycles (waveform: sine wave) at a rate of 5 Hz according to the load levels presented in **Figure S4**.


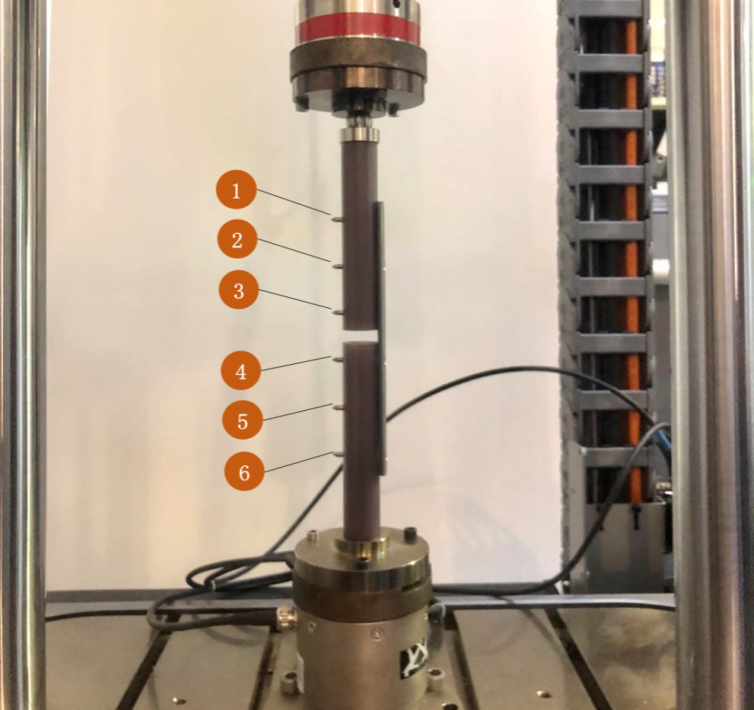


**Figure S3 High-cycle dynamic fatigue testing on the testing machine.**


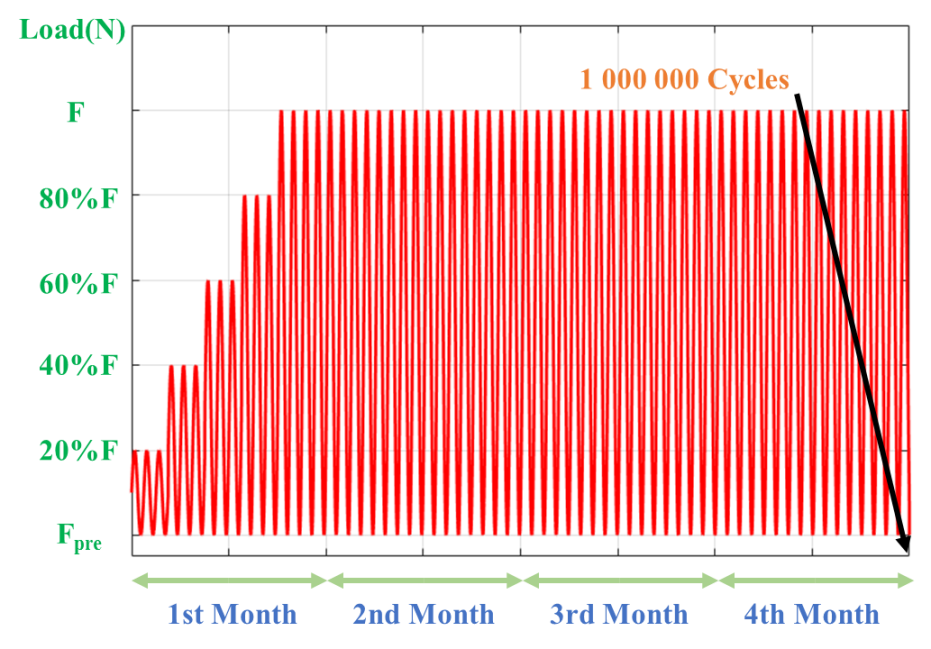


**Figure S4 Progressive dynamic loading protocol:**(20%, 40%, 60%, 80%, and 100% of designed weight expressed by F) for dynamic cycling with 1,000,000 cycles to approximate a three-to-four-month testing period, after application of a preload.

**2.2** **Defect detection:** **micro-nano tomography**

All samples were carefully collected, sorted, and cleaned, and then micro-nano tomography was performed to observe the damage location and damage mode of the samples under high cycle fatigue testing. First, we took a picture of the sample and measure the maximum size. The maximum size determines the scanning parameters and resolution, and the resolution can be set through the software (as shown in **Figure S5)**. For samples of different sizes, the resolution needs to be determined by the diameter of the field of view and the image matrix (pixels), such as 10XL, which means the diameter of the field of view is 10mm, XL means the pixel is 2048X2048, and its resolution is 10mm/2048=4.88μm , another example is 50L, which means that the diameter of the field of view is 50mm, and L means that the pixel is 1028X1028, and its resolution is 50mm/1028=48.64μm. After the sample size is determined, scan the sample and adjust the position of the radiation source and detector so that the sample testing part is in the center of the field of view (as shown in **Figure S5**). Equipment name: micro-nano tomography scanner. Device model: TomoScope L 300. Device number: MDTC-EQ-M52-01. Verification/calibration valid until: November 15, 2021. Environmental conditions: Temperature: 22.5℃-25; Humidity: 20%RH-29. Reference standard: GB/T 37166-2018, Nondestructive Testing, Computed Tomography (CT) Testing Methods for Composite Materials Industry. The test parameters used are shown in **Table S2.**


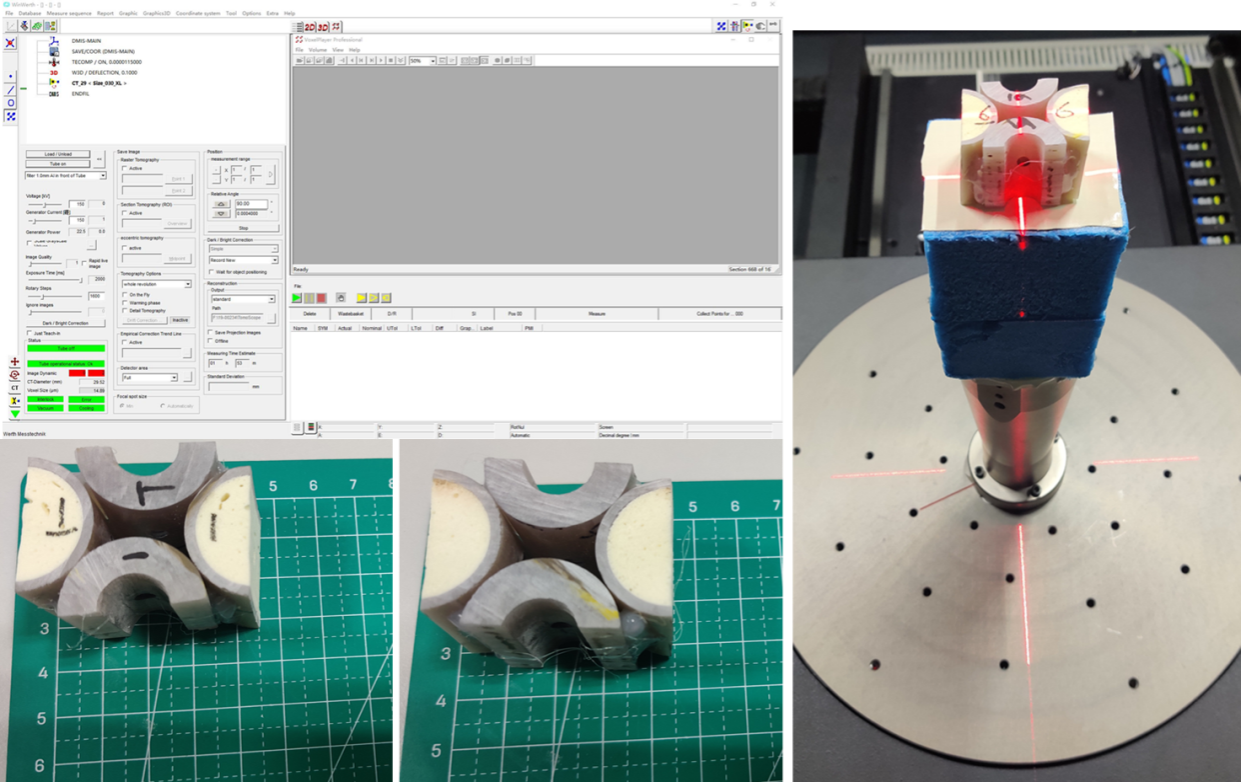

**Figure S5 Defect detection with micro-nano scanning:** Set scan parameters and resolution through software.

Table S2 Scan parameters of micro-nano tomography

|  | FCL construct Bone surrogate | SWL construct  Bone surrogate | LP construct  Bone surrogate |
| --- | --- | --- | --- |
| Ray source type | Transmission  X-ray source | Transmission  X-ray source | Transmission  X-ray source |
| Tube voltage (kV) | 150 | 200 | 200 |
| Tube current (mA) | 0.15 | 0.15 | 0.12 |
| Detector type | Array detector | Array detector | Array detector |
| Field of view diameter (mm) | 29.52 | 29.52 | 49.19 |
| Spatial resolution (μm) | 14.89 | 14.89 | 49.63 |
| Sampling time (ms) | 2000 | 2000 | 1000 |
| rear collimator size | 5mm×5mm | 5mm×5mm | 5mm×5mm |
| (width x height) |
| Image Quality | 1 | 1 | 1 |
| Turntable rotation steps | 1600 | 1600 | 1000 |
| Filter | 1.0 mm Al | 1.0 mm Al | No filter |
| scanning method | Cone beam scanning | Cone beam scanning | Cone beam scanning |
| Image matrix | 2048×2048 | 2048×2048 | 1024×1024 |
| each test piece | 1h52~54mins | 1h53~54mins | 1h10mins |

**3. Implants manufactured by 3D printing as a proof-of-concept (Shown in Figure 3C)**

All samples were printed in titanium (TI-6AL-4V) on a Renishaw AM 400 metal 3D printer (Renishaw plc, UK). System forming area size: 250mm x 250mm x 300mm. System host international authoritative certification license: TUV, CE, ISO certification, all imported. Forming thickness: 0.02-0.05mm, adjustable. Laser type: 400W fiber laser, analog pulsed. Laser beam diameter 70µm. Vacuum Assist System: Guaranteed. Oxygen control: an inert gas regulation system with oxygen content monitoring, according to the forming environment requirements for low-oxygen control of aluminum alloys and titanium alloys, the oxygen content must be as low as 100PPM within 1 hour of system startup to guaranteed forming quality and operational safety of reactive metals such as titanium and aluminium alloys. Operating gas: argon, to ensure better quality of parts, all materials use argon, other manufacturers use nitrogen to process stainless steel materials, but titanium alloys and aluminum alloys must use argon. Forming accuracy: repeatability is ±50μm. X,Y axis scanning speed: 2m/s; X,Y positioning speed, 7m/s.

**3. Safety assessment results of the three constructs**


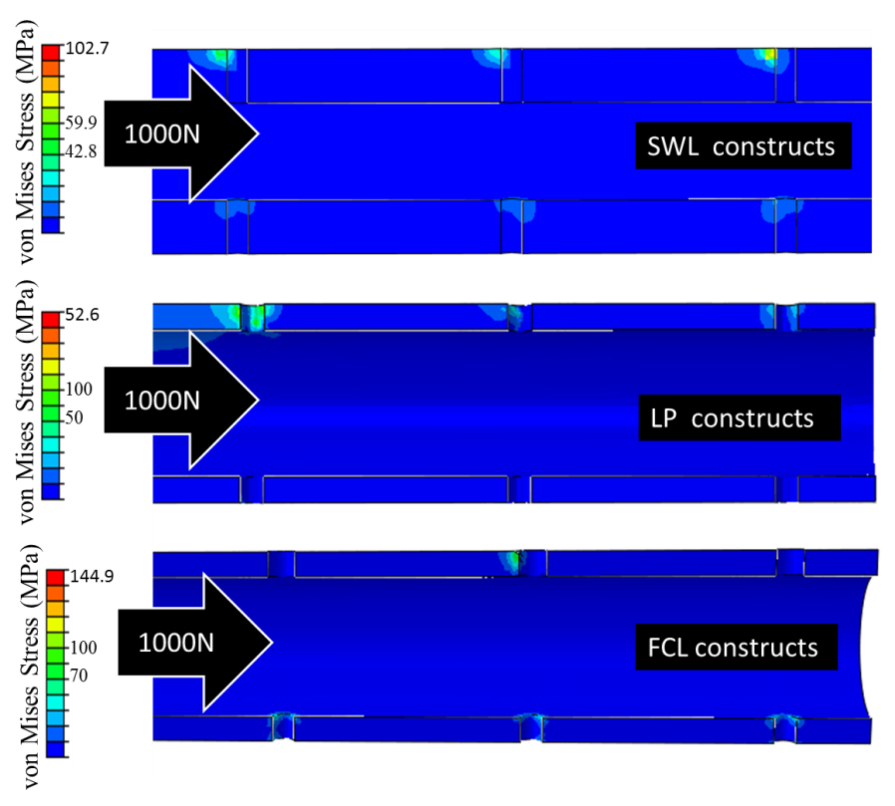


**Figure S6** The stress distribution cloud diagram of the bones of the three constructs under the axial compressive loading condition (1000N).


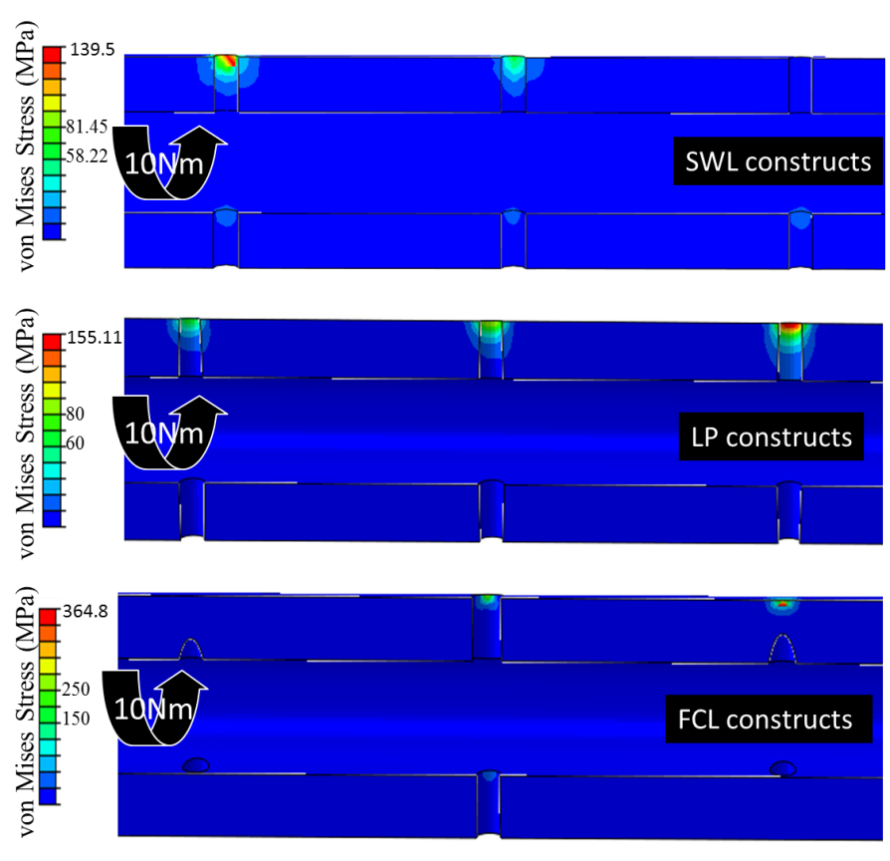


**Figure S7** The stress distribution cloud diagram of the bones of the three constructs under torsional loading conditons (10Nm).

1. **Supplementary description of boundary and loading conditions**

Model establishment was based on axial compression tests through a proximal sphere(rigid clamp), replicating the axial loading scenario of the bench-top test(Figure S8, a). The distal ends of the bone models were fully constrained as boundary condition. Torsion was applied around the diaphyseal shaft axis (Figure S8, b). In LP constructs, screws were assumed fully bonded to the bone and the plate using tie constraint. In FCL and SWL constructs, screws were bonded to the far cortical bone and the plate using tie constraint and assumed relative motion between the screws and near cortical bone using frictional sliding contact (a standard Coulomb friction coefficient of 0.3 was employed based on some of the recent studies).

For the static loading simulations, construct stiffness in non-osteoporotic bone surrogates was assessed under axial compression and torsion by loading to 1 kN and 10 Nm, respectively. In addition to actuator displacement (displacement of the center of mass of the proximal sphere), interfragmentary motion under axial compression was recorded at the near and far cortices. Axial stiffness was calculated by dividing the axial load amplitude by the actuator displacement amplitude. Torsional stiffness was calculated by dividing the torsion amplitude by the amplitude of rotation (α) around the diaphyseal axis. Torsional stiffness was multiplied by the unsupported specimen length to derive torsional rigidity.


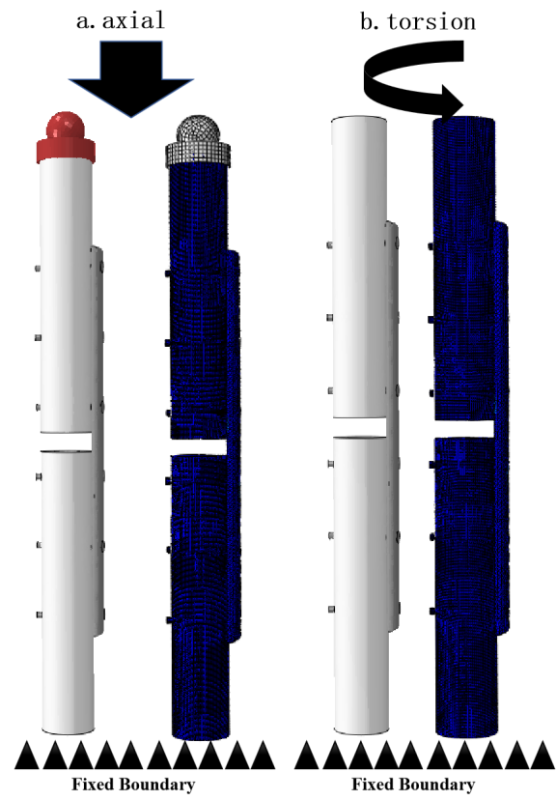


**Figure S8** Geometric model diagram and grid model diagram of numerical model. Construct stiffness were evaluated under two loading conditions: axial compression (a), torsion (b).

1. **Supplementary description of meshing**

The plates, the bone surrogates models, and screws were meshed with structured grid in Altair HyperWorks® (Troy, Michigan, USA). The choice of the mesh element ensured successful meshing of complex geometries with reasonable element quality. The finite element model of each construct in this study was calculated and analyzed by using the structural mesh C3D8R. The mesh independence was discussed here, and the maximum von Mises stress of bones and screws was used as a standard to demonstrate. By creating a mesh model with five different cell seed sizes to calculate the mesh independence from the calculation results, the mesh does not affect the calculation results when increasing the mesh size produces about 5% or less variation in the results, That is, the grid independence condition is satisfied. Based on the convergence test (Table S3,S4), the element size of the bone was 0.8mm, and the element size of the screw was 0.4mm (within 5% difference in percentage) as the mesh size of the model element.

| Table S3 Mesh sensitivity test of far cortical locking construct | | | | | | | | |
| --- | --- | --- | --- | --- | --- | --- | --- | --- |
| Meshing scheme | Plan 1 | | Plan 2 | | Plan 3 | | Plan 4 | Plan 5 |
| Bone mesh size（mm） | 2 | | 1.6 | | 1.2 | | 0.8 | 0.6 |
| Bone element number | 8568 | | 13240 | | 37476 | | 121320 | 280752 |
| Screw mesh size（mm） | 1 | | 0.8 | | 0.6 | | 0.4 | 0.2 |
| Screw element number | 1168 | | 1488 | | 3228 | | 8740 | 64576 |
| computing time | 7mins 28s | | 9mins 14s | | 26mins19s | | 1h 39mins | 11h 27mins |
| Maximum bone  Von Mises stress (MPa) | 73.72 | | 66.10 | | 112.4 | | 144.9 | 147.7 |
| Stress variation rate（%） | -11.53% | | 41.19% | | 22.43% | | 1.90% | - |
| Maximum screw  Von Mises stress (MPa) | 493.2 | | 475.9 | | 513.5 | | 574.9 | 575.8 |
| Stress variation rate（%） | -3.64% | | 7.32% | | 4.34% | | 1.07% | - |
|  | |  | |  | |  |  |  |
| Table S4 Mesh sensitivity test of locked plating construct | | | | | | | | |
| Meshing scheme | Plan 1 | | | Plan 2 | | Plan 3 | Plan 4 | Plan 5 |
| Bone mesh size（mm） | 2 | | | 1.6 | | 1.2 | 0.8 | 0.6 |
| Bone element number | 9440 | | | 14336 | | 32088 | 113679 | 292464 |
| Screw mesh size（mm） | 1 | | | 0.8 | | 0.6 | 0.4 | 0.2 |
| Screw element number | 1168 | | | 1488 | | 3328 | 8740 | 64576 |
| computing time | 21mins 48s | | | 23mins 8s | | 30mins 6s | 1h 17mins | 4h 56mins |
| Maximum bone  Von Mises stress (MPa) | 30.59 | | | 34.54 | | 43.37 | 52.60 | 55.36 |
| Stress variation rate（%） | 11.44% | | | 20.36% | | 17.55% | 4.99% | - |
| Maximum screw  Von Mises stress (MPa) | 112.8 | | | 118.5 | | 128.7 | 138.0 | 158.9 |
| Stress variation rate（%） | 4.81% | | | 7.93% | | 17.71% | 1.57% | - |
|  | |  | |  | |  |  |  |

|  | | | |
| --- | --- | --- | --- |
| Table S5 The peak von Mises stress (MPa) of plates of the LP constructs and FCL constructs under axial compression (0 to 1000N) | | | |
| Axial load（N） | LP constructs |  | FCL constructs |
|  | Plate |  | Plate |
| 100 | 6.269 |  | 10.12 |
| 200 | 12.54 |  | 20.24 |
| 300 | 18.82 |  | 30.36 |
| 400 | 25.11 |  | 40.48 |
| 500 | 31.40 |  | 50.59 |
| 600 | 37.69 |  | 60.70 |
| 700 | 43.99 |  | 67.84 |
| 800 | 50.30 |  | 73.41 |
| 900 | 56.61 |  | 78.67 |
| 1000 | 62.93 |  | 84.02 |

|  | | | |
| --- | --- | --- | --- |
| Table S6 The peak von Mises stress (MPa) of plates of the LP constructs and FCL constructs under Torsion (0 to 10Nm) | | | |
| Torsion（Nm） | LP constructs |  | FCL constructs |
|  | Plate |  | Plate |
| 1 | 14.97 |  | 16.44 |
| 2 | 29.94 |  | 32.85 |
| 3 | 44.92 |  | 47.24 |
| 4 | 59.90 |  | 59.08 |
| 5 | 74.89 |  | 72.17 |
| 6 | 89.88 |  | 85.67 |
| 7 | 104.2 |  | 99.23 |
| 8 | 119.9 |  | 112.9 |
| 9 | 134.9 |  | 126.7 |
| 10 | 149.9 |  | 140.5 |

**6. Supplementary description of the safety assessment**

Results that met our first two optimization objects (controllable two-phase stiffness and nearly parallel interfragmentary motion) were output as the object of the safety assessment. Finally, the result of optimization through the safety assessment represented the new constructs. There are two main aspects of the safety assessment: structural strength analysis based on the allowable stress, and high-cycle fatigue numerical analysis.

**The allowance stress** of the titanium alloy was adopted for safety assessment, following previously published work (Deng et al. 2021). The allowable stress is defined as the yield stress divided by a safety factor (Badalassi et al. 2014; Casavola et al. 2011). The structure von Mises stress under complex stress conditions should be less than the allowable stress, as expressed by:

|  | $Von Mises Stress=\sqrt{\frac{1}{2}\left[ \left( \sigma_{1}-\sigma_{2} \right)^{2}+\left( \sigma_{2}-\sigma_{3} \right)^{2}+\left( \sigma_{3}-\sigma_{1} \right)^{2} \right]}\leq\left[ \sigma\right]=\frac{\sigma_{Y}}{n}$ | （3） |
| --- | --- | --- |

where $\sigma_{Y}$ is the yield strength; $n$ is the safety factor, which is based on several considerations such as the accuracy of predictions on the imposed loads, strength, wear estimates, and the environmental effects to which the product will be exposed in service Bottlang et al. (2014); and $\left[ \sigma\right]$ is the allowable stress. The maximum stress of the structure should be less than the allowable stress and can be shown as:

|  | $\sigma_{max}<[\sigma]$ | （4） |
| --- | --- | --- |

The yield strength of the titanium alloy is 825 MPa. The allowable stress method was used to design the implant and evaluate the safety of the construct, so the factor of safety $n$ should greater than 2, according to structural design specifications (Bottlang et al. 2010).

**High-cycle fatigue numerical analysis** was performed in a commercial fatigue analysis program (Fe-safe®, Safe Technology, Sheffield, UK). The fatigue properties of the materials were described by fatigue strength (S) - cycle times (N). The fatigue tests were run using a sinusoidal cyclic load waveform at a constant frequency of 5 Hz in axial compression load. The tests were performed until failure occurred or until 1,000,000 cycles were completed, which corresponded to approximately a three- to four- month testing period (Bottlang et al. 2010; Claes et al. 1998). Progressive dynamic loading based on the designed load F = 1000 N is shown in **Figure. S4**. The safety factor of the structure is effectively evaluated above. This stress ratio was referred to ISO 14801 to simulate the condition of physiological loading. Brown–Miller criteria with Morrow mean stress correction was used for the lifetime calculation. This algorithm used the strain-life curve defined by Uhthoff et al. (2006):

|  | $\frac{\gamma}{2}\text{+}\frac{\Delta\varepsilon_{N}}{2}=1.65\frac{\sigma_{f}^{'}-\sigma_{N,m}}{E}\left(2N_{f}\right)^{b}+1.75\varepsilon_{f}^{'}\left(2N_{f}\right)^{c}$ | （5） |
| --- | --- | --- |

where $\sigma_{N,m}$is the mean normal stress on the screw, $2N_{f}$is the number of reversals to crack initiation, $\frac{\gamma}{2}$ is the shear strain amplitude, $\Delta\varepsilon_{N}$ is the normal strain on the screw, $\sigma_{f}^{'}$is the fatigue strength coefficient, $\varepsilon_{f}^{'}$ is the normal fatigue ductility coefficient, $E$ is the elastic modulus, $c$ is the fatigue ductility exponent, and $b$ is the fatigue strength exponent. The fatigue safety coefficient $m$ can be used to describe the safety under the specific fatigue environment:

|  | $m>1$ | （6） |
| --- | --- | --- |
|  |  |  |

**7. Supplementary description of the response surface model** **(RSM) method**

The matrix form of the RSM can be shown as:

|  | $\boldsymbol{y}=\boldsymbol{X}\beta+\boldsymbol{\varepsilon}$ | （7） |
| --- | --- | --- |

Where X means the optimization parameters ($E,\gamma, t, d$), Y means the optimization objective ($K, \delta, \sigma$) in our model. β is the matrix of coefficients, ε is the residual matrix.

$\boldsymbol{y=}\left[ \begin{aligned} \boldsymbol{y}_{\boldsymbol{1}} \\ \boldsymbol{y}_{\boldsymbol{2}} \\ \boldsymbol{\vdots} \\ \boldsymbol{y}_{\boldsymbol{n}} \end{aligned} \right]$**,$\boldsymbol{X=}\left[ \begin{aligned} \boldsymbol{1}\boldsymbol{x}_{\boldsymbol{11}}\boldsymbol{x}_{\boldsymbol{12}}\boldsymbol{\cdots}\boldsymbol{x}_{\boldsymbol{1k}} \\ \boldsymbol{1}\boldsymbol{x}_{\boldsymbol{21}}\boldsymbol{x}_{\boldsymbol{22}}\boldsymbol{\cdots}\boldsymbol{x}_{\boldsymbol{2k}} \\ \boldsymbol{\vdots\vdots\vdots\ddots\vdots} \\ \boldsymbol{1}\boldsymbol{x}_{\boldsymbol{n1}}\boldsymbol{x}_{\boldsymbol{n2}}\boldsymbol{\cdots}\boldsymbol{x}_{\boldsymbol{nk}} \end{aligned} \right]$,** $\beta=\left[ \begin{aligned} \beta_{1} \\ \beta_{2} \\ \vdots\\ \beta_{n} \end{aligned} \right]$*,* $\boldsymbol{\varepsilon=}\left[ \begin{aligned} \boldsymbol{\varepsilon}_{\boldsymbol{1}} \\ \boldsymbol{\varepsilon}_{\boldsymbol{2}} \\ \boldsymbol{\vdots} \\ \boldsymbol{\varepsilon}_{\boldsymbol{n}} \end{aligned} \right]$ （8）

The minimum variance was:

|  | $\boldsymbol{L}=\sum_{i=1}^{n} \varepsilon_{i}^{2}=\boldsymbol{\varepsilon}^{\boldsymbol{'}}\boldsymbol{\varepsilon}={(\boldsymbol{y}-\boldsymbol{X}\beta)}^{'}(\boldsymbol{y}-\boldsymbol{X}\beta)$ | （9） |
| --- | --- | --- |

When the minimum variance was at its lowest value, it was clear that the fitted surface was closest to the true value. When the partial derivative of L with respect to β was zero, the minimum variance took the minimum value.

|  | $\frac{\partial\boldsymbol{L}}{\partial\beta}\vert_{\hat{\beta}}=-2\boldsymbol{X}^{\boldsymbol{'}}\boldsymbol{y}+2\boldsymbol{X}^{\boldsymbol{'}}\boldsymbol{X}\hat{\beta}=0$ | （10） |
| --- | --- | --- |

Thereby:

|  |  | （11） |
| --- | --- | --- |

The fitted response surface can be shown as:

|  | $\hat{\boldsymbol{y}}=\boldsymbol{X}\hat{\beta}$ | （12） |
| --- | --- | --- |
